# The Improvement of Idiots

**Published:** 1889-06-08

**Authors:** 


### The Improvement of Idiots.

To many people the idea of an idiot is more unpleasing than that of a lunatic. In the latter there is intelligence, though misdirected; in the former there is little or none. For the lunatic one feels a certain respect amid our pity; for the idiot there can be only pity felt by the most tender heart, and by hearts that are not tender a certain contempt and loathing. The lunatic, driven distraught by some real sorrow, or even by some imaginary terror, is a tragic figure. Lear and Ophelia touch us on the nobler side of our nature. But only Wordsworth, who was at times the most prosaic of great poets, has ventured to deal with idiocy; and it is only by courtesy that the story of Betty Fry and her idiot son can be called poetry at all, while what pathos dwells in it lies with the mother's love, not with the child's condition. In this distinction literature echoes the feelings of humanity. The "sweet bells, jangled out of time and harsh," have still some lingering remnants of their former charm, but there is none in those poor cracked instruments that at best could give no better sound than an infant's rattle.

But can nothing be done for these poor weak intellects that cannot hold their own among their brethren? The earthen pipkin may not be fit to swim with the iron pots, but it has uses of its own. Our asylums have improved; the chains and whip have disappeared from them, and "Bedlam" has returned to the older form of "Bethlem," with a great change in the meaning of the word. Similarly, a change has taken place in the treatment of idiots. It is not assumed that because their intelligence is somewhat less than that of their fellows, they are altogether to be debarred from either usefulness or pleasure in life. A visit to Earlswood would clear away many cobwebs from the brains of those who use the name of the asylum as a synonym for helpless imbecility, even though it might make them think that there were more "candidates for Earlswood" going loose about the world than they had fancied.

Let it be admitted that those to whom Earlswood gives shelter are just so much weaker than average humanity that they cannot be trusted to walk unassisted through a troublesome and not too honest world. It does not follow from this that they have no faculty for useful work, and cannot, under kind and careful guidance, justify their existence in the eyes of God, if not of man. Earlswood is a partially self-supporting institution, supported by the work of its inmates. At least half the male patients, and somewhat less than half the female ones, are able to follow some handicraft, and thus, armed with some means of earning their bread, many are able to return to their homes, no longer the useless burdens that they were before. The shoemaking, tailoring, and printing, as well as much of the carpentering, is done by inmates, while in the mat-making industry they are able to compete in the public markets.

Even those who are denied the blessing of useful work are not altogether pitiable. The very limits of their intelligence make them happy. They are pleased with trifles, overjoyed at a word of praise, as children are. They are not troubled, as so many of the insane are, with terrors of a glowing after-world or distrust of their fellow-men; the very limits of their intelligence form a protecting wall of happiness around them. They are children—children for all time, and will grow to full life only in the sunshine of eternity—but like children they are bright and cheerful; like children they have within them dormant faculties that may be trained to use.

